# Long-term lifestyle interventions in middle-aged and elderly men with nonalcoholic fatty liver disease: a randomized controlled trial

**DOI:** 10.1038/srep36783

**Published:** 2016-11-10

**Authors:** Fangyuan Dong, Yan Zhang, Yiqin Huang, Yiqian Wang, Gansheng Zhang, Xiaona Hu, Jiaofeng Wang, Jie Chen, Zhijun Bao

**Affiliations:** 1 Department of Gastroenterology, Huadong Hospital, Shanghai Medical College, Fudan University, No. 221 Yan’an West Road, Shanghai 200040, P.R. China; 2 Shanghai Key Laboratory of Clinical Geriatric Medicine, No. 221 Yan’an West Road, Shanghai 200040, P.R. China; 3 Research Center on Aging and Medicine, Fudan University, No. 221 Yan’an West Road, Shanghai 200040, P.R. China

## Abstract

Nonalcoholic fatty liver disease (NAFLD), a metabolic disorder related to insulin resistance and metabolic syndrome, has become a public health concern. Currently, the principal therapeutic modalities targeting NAFLD are lifestyle interventions. However, the efficacy of long-term lifestyle interventions in managing NAFLD remains largely unexplored. This study aimed to evaluate the efficacy of long-term lifestyle interventions in middle-aged and elderly men with NAFLD. All 280 eligible patients were randomized to the control or test group. Patients in the test group received counseling on diet and exercise from 2 physicians every 3 months via a phone call. Patients in the control group received only counseling in annual checkups without regular intervention. After the 2-year periodic intervention, body weight, abdominal circumference, ALT, TCH, LDL-C and HDL-C decreased in the test group. Specifically, the fatty liver index (FLI) and NAFLD-fibrosis score (NAFLD-FS) reduced markedly in the test group. However, in the control group, there was only a significant decrease in LDL-C, HDL-C and NAFLD-FS (*P* < 0.001). The liver steatosis grade of the test group decreased significantly, while it increased in the control group. In NAFLD, long-term lifestyle interventions exert an anti-obesity effect and attenuate liver dysfunction and steatosis.

Nonalcoholic fatty liver disease (NAFLD) is a term encompassing a spectrum of clinical liver abnormalities ranging from simple hepatic steatosis without biochemical liver dysfunction to steatohepatitis and even cirrhosis and hepatocellular carcinoma^1^,^2^. NAFLD has become a global health hazard in parallel with the obesity pandemic. The prevalence of NAFLD ranges from 15% to 35% in the general population in Europe and the Middle East, with similar numbers in China, and increases to as high as 70% in obese individuals and 90% in diabetics^3^,^4^. Several risk factors contribute to the development of NAFLD, including obesity, sedentary lifestyles and high-calorie diets. In addition to the metabolic syndrome components such as obesity, hypertension, diabetes, and lipid metabolic disorder, the pathogenesis of NAFLD is also closely related to gender and age^5^.

In patients with nonalcoholic steatohepatitis, half of deaths are due to cardiovascular diseases and malignancies, yet awareness of these risks remains low^6^. Cirrhosis, the third leading cause of death in patients with NAFLD, is predicted to become the most common indication for liver transplantation^2^. The incidence of NAFLD-related hepatocellular carcinoma is increasing, and up to 50% of cases may occur in the absence of cirrhosis^7^. As such, the treatment of NAFLD has become an area of much interest. Non-pharmacological lifestyle interventions positively affect NAFLD and are recommended as a first-line therapeutic method. Diet and exercise are the main treatment for the majority of patients with NAFLD. Weight loss is beneficial, and the degree of liver histological improvement is proportional to the amount of weight loss^8^,^9^. However, most individuals fail to achieve proper weight control, and compliance with lifestyle modifications is typically poor. Therefore, we examined whether appropriate guidance regarding diet and exercise offered periodically would improve the state of NAFLD. According to a meta-analysis conducted by Lorenzo A. *et al*.^10^, exercise-based interventions for NAFLD can reduce intrahepatic lipid content and attenuate hepatocellular injury; unfortunately, most trials involved lasted less than one-year.

To better understand this issue, we performed a large-scale, randomized controlled trial to investigate the impact of long-term lifestyle interventions on middle-aged and elderly men with NAFLD as demonstrated by liver ultrasound.

## Results

### Baseline characteristics of the participants

**Figure 1:**
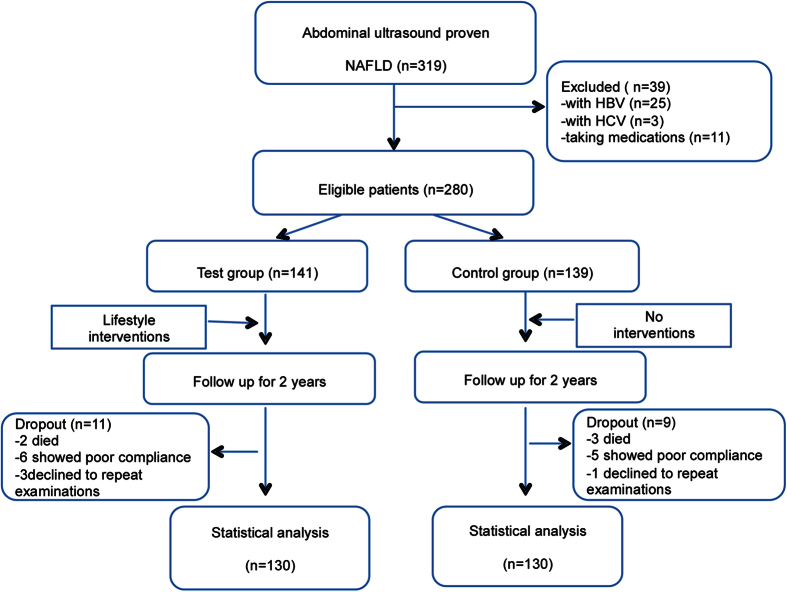
Flowchart of the trial. Patients in the control group did not receive any lifestyle intervention, whereas patients in the test group received periodic diet and exercise guidance from 2 experienced physicians. A total of 260 patients completed the longitudinal follow-up.

A total of 319 patients were enrolled in this study. Notably, 25 patients with hepatitis B, 3 with hepatitis C and 11 taking medications were removed. During the first and the second year follow-ups, 8 and 12 patients dropped out of the study, respectively (2 died of cardiovascular diseases, 2 died of cancers and 1 died of a car accident; 11 showed poor compliance and 4 were reluctant to repeat physical examinations). Thus, a total of 260 subjects were included in the final analysis (130 in the test group and 130 in the control one). Overall, the average age of the subjects was 57.31 ± 5.55 years. No significant difference was observed in the baseline parameters between the two groups (see in Table 1). A consort flow diagram of this study is shown in Fig. 1.

### Alterations in physical parameters

In parallel with the significant decrease in body weight and abdominal circumference (*P* < 0.001), laboratory variables (including ALT, TCH, LDL-C and HDL-C) improved significantly (*P* < 0.001) in the test group; the serum TG concentration almost reached the normal range, but this improvement did not achieve statistical significance. Furthermore, we observed a significant decrease in the FLI and NAFLD-FS in the test group. However, in the control group, there was a clear improvement in only LDL-C, HDL-C and NAFLD-FS (*P* < 0.001), whereas the other parameters (including body weight, abdominal circumference, ALT, TCH, TG, and FLI) remained essentially unchanged. Within the test group, there was an apparent effect on body weight and dyslipidemia before and after the intervention. However, when compared with the control group at the end of follow-up, these variables did not show significant discrepancies (see Tables 2 and 3).

### Alterations in liver fat deposition

**Figure 2:**
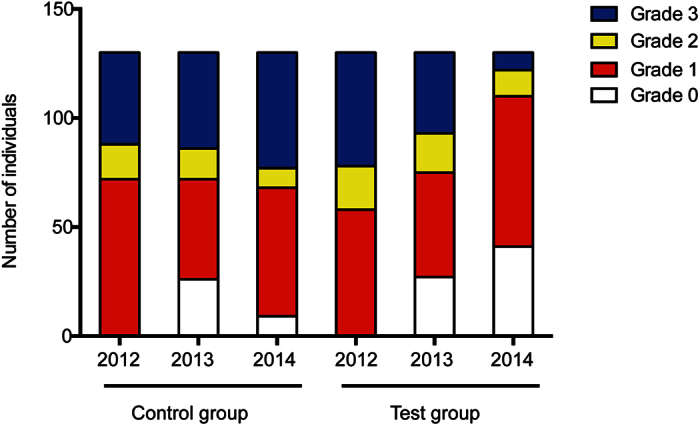
Changes in liver steatosis grade detected by liver ultrasound.

The changes in liver steatosis grade detected by liver ultrasound are summarized in Fig. 2. At entry, neither the individual hepatic steatosis grade nor the proportion of patients distributed in the four categories significantly differed between the two groups. After the first year of the intervention, an improvement in liver ultrasound results was found in both groups, but no significant difference in liver fat deposition was observed. After the second year of the intervention, however, the hepatic steatosis grade of the test group was clearly reduced, whereas it increased in the control group. The number of grade 1 steatosis patients increased dramatically in the test group, while the number of grade 3 patients grew significantly in the control group (*P* < 0.001). The results are summarized in Tables 2 and 3.

### Adverse Effects

The lifestyle intervention was well tolerated. No adverse events associated with the lifestyle modification program were reported for any cases during the entire trial, due to the appropriate guidance provided and the good adherence to the intervention.

## Discussion

NAFLD, a hepatic manifestation of the metabolic syndrome, has become a common but often silent chronic liver disease worldwide. Insulin resistance and oxidative stress are involved in the pathogenesis of NAFLD^11^. Lifestyle interventions focusing on diet and exercise remain the cornerstone of NAFLD treatment. Several studies have found that gradual weight loss and regular physical activity can help treat NAFLD by improving insulin sensitivity and reducing hepatic steatosis^12^,^13^. In this prospective study, a two-year lifestyle intervention of a diet tailored to individual calorie requirements and an increase in physical activity was associated with a significant improvement in the severity of hepatic steatosis and liver dysfunction.

The peak prevalence of NAFLD has been reported to be in those 50 to 65 years old. Additionally, the prevalence of NAFLD was significantly higher in males than in females within the same age group^14^. Furthermore, men in this age bracket often develop bad lifestyle habits such as sedentary behavior and high-fat dietary patterns. However, women in this age group usually experience menopause, and thus the prevalence of NAFLD tends to increase partly due to the decreases in estrogen. Therefore, we chose to investigate male participants who were older than 45 years old in this study. Improvements in liver dysfunction by lifestyle intervention are considered to be a consequence of the restoration of insulin sensitivity. Accumulating evidence suggests that NAFLD is associated with obesity, diabetes mellitus and dyslipidemia. A primary study found that weight changes were markedly correlated with changes in biochemical parameters. Additionally, weight loss may be a predictor of all non-alcoholic steatohepatitis-related histologic improvements^15^,^16^. Consistent with previous findings, our results demonstrated that a 2-year periodical lifestyle intervention based on diet control and increases in physical activity in middle-aged and elderly men with NAFLD decreased liver enzyme, TCH, LDL-C and HDL-C levels, body weight and abdominal circumference. Moreover, it should be noted that the lifestyle intervention achieved a significant degree of reduction in hepatic steatosis.

The strengths of the present study included the recruitment of a consecutive series of a large number of subjects and the low dropout rate (7.1%), which minimized the selection bias. Another advantage was the duration of the long-term longitudinal follow-up, as it surpassed those of most similar studies.

In interpreting our findings, several major limitations need to be considered. First, we diagnosed NAFLD through ultrasound rather than the gold standard liver biopsy. Ultrasound is an inexpensive and noninvasive diagnostic tool that has a sensitivity of 93% when there is more than 33% steatosis; however, the sensitivity is poor when there is less than 30% steatosis^17^,^18^. Additionally, ultrasound cannot detect liver inflammation or fibrosis. Newer ultrasound techniques that can more accurately quantify hepatic fat content may partly overcome this limitation^19^. According to the American Association for the Study of Liver Diseases (AASLD) guidelines, liver biopsy is the gold standard for diagnosing NAFLD^20^. However, due to its invasiveness and possible complications, most subjects are not inclined to accept a biopsy to diagnose NAFLD. Other methods such as computed tomography (CT), magnetic resonance imaging (MRI) and Fibroscan are also recommended for diagnosing NAFLD. CT does not improve the detection sensitivity if steatosis is mild and has the disadvantages of increased cost and radiation exposure. MRI, including magnetic resonance spectroscopy (MRS), is able to detect the presence of hepatic fat greater than 5.56% with an accuracy rate close to 100%; however, both are expensive, and MRS has limited availability^21^. Perhaps we can employ liver biopsy, Fibroscan CAP or MRI to improve the detection accuracy in further studies. Second, because the data were obtained from outpatients, only partial data on fasting insulin, TNF-α, IL-6, adiponectin and leptin were collected. Hence, we did not show these data in our results. Third, the intensity of our intervention was insufficient. A retrospective study compared mild-, moderate-, and high-intensity exercise regimens in 169 patients with detectable hepatic fat. Only participants in the highest intensity exercise group (>250 min/week) had improved metabolic parameters and significant hepatic fat reduction^22^. Previous research demonstrated that the decrease in serum ALT levels was associated with the amount of weight loss (r = 0.35). The AASLD practice guidelines indicate that weight loss of least 3–5% appears necessary for improving steatosis, but a greater loss (up to 10%) may be needed to alleviate necroinflammation^23^. A recent study showed that intensive inpatient and ambulatory treatments are more effective than usual care^24^. According to Vilar-Gomez *et al*.^25^, aggressive lifestyle interventions can resolve NASH and regress fibrosis. Additionally, we did not involve resistance training in our study. Indeed, there is evidence that both aerobic activity and resistance training have beneficial effects on NAFLD. In a large sample of Korean adults, it appeared that any reasonable amount of physical activity was better than no physical activity because prolonged sitting times were shown to be positively associated with the prevalence of NAFLD^26^. Lastly, our records were not very detailed regarding the components of the participants’ diet and exercise. Therefore, additional studies with thorough designs are needed to confirm previous findings.

As the use of repeated liver biopsy may not be possible for ethical and practical considerations, it was difficult to obtain liver biopsy as well as some serological indicators (such as fasting insulin and TNF-α) for all the subjects. However, we believe that the repeatability and practical utility of the lifestyle intervention is worthwhile. In the future, we plan to popularize the intervention using the media. Certainly, for patients with NASH, a lifestyle intervention alone is far from sufficient; supplementary beverages, foods and drugs are essential to alleviating liver inflammation and necrosis.

Several polyphenols, such as curcumin, anthocyanins, resveratrol and those present in coffee, tea, and soy, are available in the diet; their consumption can be proposed as part of a healthy diet for NAFLD treatment. Natural phenolic compounds can promote lipolysis, inhibit lipogenesis and counteract hepatic fibrogenesis^27^. In a cross-sectional study, although no association was demonstrated between coffee consumption and the new onset of NAFLD, high consumption of coffee was related to a lower proportion of clinically significant fibrosis ≥ F2; part of the benefits can be attributed to caffeine content^28^,^29^. Experimental studies have shown that diets enriched with omega-3 polyunsaturated fatty acid can increase insulin sensitivity in rats, reduce intrahepatic triglyceride levels and improve steatohepatitis^30^.

Notably, insulin sensitizers such as metformin play a positive role in NAFLD treatment^31^. Several other medicines such as probiotics and vitamin A and E have also been reported to be efficient in managing NAFLD^32^,^33^,^34^. However, the effects of pharmacological agents on NAFLD require further exploration.

In conclusion, the present study demonstrates that a 2-year lifestyle intervention for middle-aged and elderly men with NAFLD manifests an anti-obesity effect and reduces liver dysfunction and steatosis. The end point of our study included changes in body weight, liver enzymes and intrahepatic lipid content. Based on the longitudinal follow-up of patients with NAFLD and a periodical oral intervention, the severity of liver steatosis decreased and related metabolic indicators improved. Without the intervention, the prevalence of fatty liver had a progressive trend. The effect of verbal intervention alone is limited, but as it is easy to implement and convenient to communicate via media, it will play a crucial role in the general population with NAFLD. The amount of physical activity per week was essential to the overall success in weight loss and maintenance. Currently, physical exercise appears to be related to a decline in hepatic fat, even in the absence of weight loss. Furthermore, patients should be encouraged to consciously sustain an appropriate diet combined with increased physical activity even when the study has been completed. Finally, our findings support the current recommendation that lifestyle modifications incorporating diet and exercise should always be the fundamental therapeutic intervention used for NAFLD. This study reinforced the notion that appropriate diets coupled with exercise could produce weight loss as well as histological improvements in NAFLD. However, more extensive investigations are urgently needed in the future.

## Methods

### Ethics Statement

This present study was approved by the Ethics Committee of Huadong Hospital, Fudan University. Before the start of the study, all participants provided written informed consent. This study was registered at chictr.org (ChiCTR-IOR-16008949) in August 2016. We confirm that the study methods and reporting were conducted in accordance with the CONSORT 2010 guidelines.

### Participants

This is a single-centered, randomized, blank-controlled trial. Between January 2012 and June 2012, 319 male patients with NAFLD were referred to the outpatient department of gastroenterology, Huadong Hospital, Fudan University. Those permanently living in Shanghai who were ≥ 45 years old, were male patients, and were confirmed by liver ultrasonography to have different degrees of liver fatty change were screened for eligibility. The exclusion criteria included the following: (1) a history of alcohol intake greater than 20 g/day or 140 g/week; (2) evidence of viral hepatitis, drug-induced liver disease, autoimmune liver disease, total parenteral nutrition, genetic disorders such as Wilson’s disease, alpha-1-antitrypsin deficiency, and other conditions that could lead to liver steatosis; (3) consumption of hypoglycemic, hypolipidemic, anti-inflammatory and weight loss agents as well as any drug known to influence liver function and the presence of coronary, renal, pulmonary and thyroid diseases; and (4) transaminase levels that were more than double the upper limit of the normal reference value.

All patients underwent routine history taking, anthropometric measurements, laboratory assessments and abdominal ultrasonography within one week prior to the beginning of the study. Participants with evidence of hepatic steatosis or abnormal blood tests of liver function were further questioned (especially regarding family history and history of drug use) and ruled out if they met the exclusion criteria. A computer-generated randomization sequence assigned the eligible patients in a 1:1 ratio to the test group with lifestyle intervention or the control group without it.

### Evaluation and monitoring of patients

All subjects were asked to avoid a high-fat diet in the evening before the examination and to fast overnight for at least 8 hours before the examination. Height and weight were measured with an accuracy of 0.1 cm and 0.1 kg, respectively. Measurements were performed in the standing position, with minimal clothing and no shoes. Fasting blood samples were collected from a cubital vein, centrifuged to separate the serum and kept at −80 °C until analysis. An ultrasound scanner (LOGIQP6 PRO, GE company) was applied to assess liver fat content. The diagnosis of NAFLD was based on the presence of at least 2 of the following 3 criteria: increased hepatic echogenicity compared to the spleen or the kidneys, blurring of liver vasculature and deep attenuation of the ultrasonic signal according to the Asia-Pacific region. Liver ultrasound was reviewed and scored by the same 2 experienced radiologists. They were blinded to treatment allocation, clinical information and laboratory data. If conflicts existed when interpreting the image, a final conclusion was determined after discussion. Hepatic steatosis was categorized as grade 0 (lack of fat accumulation), grade 1 (mild increase in echogenicity with normal visualization of the diaphragm and intrahepatic vessel borders), grade 2 (moderate increase in echogenicity with slightly impaired visualization of the diaphragm and intrahepatic vessel borders), and grade 3 (severe increase in echogenicity with markedly impaired visualization of the diaphragm, intrahepatic vessel borders, and the posterior portion of the right hepatic lobe). The baseline characteristics are illustrated in Table 1.

### Lifestyle Interventions

All enrolled patients in the test group received lifestyle counseling regarding their diet and physical activity from 2 professional physicians (one dietician and one exercise physiologist). Doctors conducted a phone visit (the duration of a typical phone visit was approximately 10 minutes) with the patients in the test group every 3 months from July 2012 to July 2014, providing health guidance on diet and exercise; in contrast, patients in the control group did not receive periodic calls concerning lifestyle interventions from their doctors. During each visit, patients in the test group were encouraged to continue adhering to the advice. Later, they were reminded to receive an annual physical examination in both July 2013 and July 2014. The detailed follow-up procedure included anthropometric measurements, collection of fasting blood samples and abdominal ultrasound as entry, as well as a face-to-face interview for all subjects. Participants could provide feedback regarding the problems they encountered and received an individual reply.

The dietary intervention aimed to control total calorie intake (especially avoiding greasy food) for ideal weight. The goal of weight management was to obtain a negative calorie balance (25–30 calories/kg/d) for overweight and obese individuals and a neutral calorie balance (30–35 calories/kg/d) for those with normal BMI. The dietary pattern was distributed as 23~30% fat (with one-third saturated and two-thirds unsaturated fatty acids), 15~20% protein, and 50~60% carbohydrate. Based on specific body mass and intensity of labor, the doctors made personalized dietary prescriptions for each individual.

Patients were advised to modify sedentary lifestyles and to choose proper exercises according to their favorite activity and physical condition. They were encouraged to engage in moderate (60~80% of target heart rate) to vigorous (≥80% of the target heart rate) aerobic exercise such as fast walking, jogging, bicycling and swimming at least 3 to 4 times per week, for 30 to 60 minutes each time. They were recommended to gradually increase their exercise time and intensity until they reached their goal and to maintain this level throughout the whole study. A simple calculation formula was used to obtain the target heart rate: (170 - age) times per minute. The duration of physical activity was monitored using self-monitoring forms.

All sessions were led by the dietician and the exercise physiologist, who provided the diet and exercise proposal. At each visit, subjects were asked to complete a three-day dietary and exercise recall and send it to the doctors by e-mail. The doctors reviewed the records to identify areas of success and areas requiring further improvement to ensure that they could communicate with patients in a timely manner. In the test group, poor compliance was determined if a patient missed two consecutive appointments and was unable to be contacted via phone, whereas in the control group, poor adherence was defined by the latter situation.

### Statistical Analysis

SPSS 21.0 software was used for the statistical analysis (SPSS Inc, Chicago, IL). Continuous variables were presented as the mean ± standard deviation. A two-tailed, paired Student’s *t*-test was applied for the comparisons between groups as well as the before and after treatment comparisons. To test the differences within the groups, Wilcoxon’s rank sum test was performed. To test the differences between the 2 groups, Mann-Whitney rank sum test was performed. All *P* values were two-sided, and values less than 0.05 were considered statistically significant.

## Additional Information

**How to cite this article**: Dong, F. *et al*. Long-term lifestyle interventions in middle-aged and elderly men with nonalcoholic fatty liver disease: a randomized controlled trial. *Sci. Rep*. **6**, 36783; doi: 10.1038/srep36783 (2016).

**Publisher’s note**: Springer Nature remains neutral with regard to jurisdictional claims in published maps and institutional affiliations.

## Figures and Tables

**Table 1:** Comparison of baseline characteristics between the test group and control group.

Parameter	Control group (n = 139)	Test group (n = 141)	*P* - value
**Anthropometric indicators**			
Age (y)	57.94 ± 5.71	56.68 ± 5.33	0.068
Height (cm)	172.00 ± 7.54	171.50 ± 6.15	0.555
Weight (kg)	75.90 ± 10.63	76.74 ± 9.88	0.511
BMI (kg/m^2^)	25.59 ± 2.58	26.04 ± 2.66	0.167
Abdominal Circumference (cm)	93.05 ± 9.41	94.14 ± 8.58	0.329
SBP (mmHg)	119.50 ± 13.72	121.92 ± 14.80	0.172
DBP (mmHg)	78.08 ± 9.09	79.65 ± 9.10	0.165
FLI	49.70 ± 23.09	51.43 ± 21.99	0.536
NAFLD Fibrosis Score	−23.80 ± 2.25	−24.18 ± 2.37	0.194
**Liver Dysfunction**			
ALT (U/L)	30.38 ± 23.00	28.65 ± 16.58	0.487
AST (U/L)	23.12 ± 12.96	22.15 ± 7.29	0.462
GGT (mmol/L)	39.59 ± 34.07	36.28 ± 22.57	0.356
**Lipid profile**			
TCH (mmol/L)	5.08 ± 0.79	5.19 ± 0.82	0.258
LDL-C (mmol/L)	3.20 ± 0.72	3.33 ± 0.76	0.158
HDL-C (mmol/L)	0.89 ± 0.17	0.93 ± 0.19	0.092
TG (mmol/L)	2.06 ± 1.18	1.91 ± 0.91	0.240
**Insulin resistance**			
FPG (mmol/L)	4.87 ± 1.17	5.00 ± 1.14	0.356
**Liver steatosis grade**			
Grade 0 n (%)	**0 (0%)**	**0 (0%)**	
Grade 1 n (%)	72 (55.4%)	58 (44.6%)	0.100
Grade 2 n (%)	16 (12.3%)	20 (15.4%)	
Grade 3 n (%)	42 (32.3%)	52 (40.0%)	

FLI = fatty liver index, NAFLD-FS = NAFLD-fibrosis score, BMI = body mass index, SBP = systolic blood pressure, DBP = diastolic blood pressure, ALT = alanine aminotransferase, AST = aspartate aminotransferase, GGT = gamma-glutamine transpeptidase, TCH = total cholesterol, TG = triglyceride, LDL-C = low-density lipoprotein cholesterol, HDL-C = high-density lipoprotein cholesterol, FPG = fasting plasma glucose.

**Table 2:** Within-group comparison of parameters in the test and control groups.

Parameters	Control group (n = 130)			Test group (n = 130)		
	Pre Intervention	After Intervention	*P* value	Pre Intervention	After Intervention	*P* value
**Anthropometric indicators**						
Weight (kg)	75.90 ± 10.63	75.97 ± 11.01	0.824	76.74 ± 9.88	75.34 ± 9.94	**<0.001**
BMI (kg/m^2^)	25.59 ± 2.58	26.01 ± 2.75	0.001	26.04 ± 2.66	25.82 ± 2.65	0.033
Abdominal	93.05 ± 9.41	94.00 ± 9.23	0.18	94.14 ± 8.58	91.79 ± 8.31	**<0.001**
**Circumference (cm)**						
SBP (mmHg)	119.50 ± 13.72	120.38 ± 12.84	0.466	121.92 ± 14.80	120.40 ± 13.70	0.235
DBP (mmHg)	78.08 ± 9.09	78.99 ± 9.41	0.314	79.65 ± 9.10	78.55 ± 11.70	0.323
FLI	49.70 ± 23.09	52.06 ± 24.38	0.152	51.43 ± 21.99	44.72 ± 23.08	**<0.001**
NAFLD Fibrosis Score	−23.80 ± 2.25	−28.85 ± 2.12	**<0.001**	−24.18 ± 2.37	−28.87 ± 2.27	**<0.001**
**Liver Dysfunction**						
ALT (U/L)	30.38 ± 23.00	28.78 ± 20.88	0.277	28.65 ± 16.58	23.95 ±12.50	**<0.001**
AST (U/L)	23.12 ± 12.96	23.56 ± 13.18	0.584	22.15 ± 7.29	21.12 ± 6.90	0.073
GGT (mmol/L)	39.59 ± 34.07	40.56 ± 32.88	0.564	36.28 ± 22.57	33.96 ± 30.39	0.216
**Lipid profile**						
TCH (mmol/L)	5.08 ± 0.79	4.86 ± 0.88	0.001	5.19 ± 0.82	4.84 ± 0.82	**<0.001**
LDL-C (mmol/L)	3.20 ± 0.72	2.85 ± 0.67	**<0.001**	3.33 ± 0.76	2.92 ± 0.68	**<0.001**
HDL-C (mmol/L)	0.89 ± 0.17	1.23 ± 0.23	**<0.001**	0.93 ± 0.19	1.27 ± 0.26	**<0.001**
TG (mmol/L)	2.06 ± 1.18	2.07 ± 1.48	0.957	1.91 ± 0.91	1.71 ± 0.93	0.009
**Insulin resistance**						
FPG (mmol/L)	4.87 ± 1.17	5.03 ± 1.29	0.162	5.00 ± 1.14	5.02 ± 1.08	0.842
**Liver Steatosis grade**						
Grade 0 n (%)	0 (0%)	9 (6.9%)		0 (0%)	41 (31.5%)	
Grade 1 n (%)	72 (55.4%)	59 (45.4%)	0.004	58 (44.6%)	69 (53.1%)	**<0.001**
Grade 2 n (%)	16 (12.3%)	9 (6.9%)		20 (15.4%)	12 (9.2%)	
Grade 3 n (%)	42 (32.3%)	53 (40.8%)		52 (40.0%)	8 (6.2%)	

FLI = fatty liver index, NAFLD-FS = NAFLD-fibrosis score, BMI = body mass index, SBP = systolic blood pressure, DBP = diastolic blood pressure, ALT = alanine aminotransferase, AST = aspartate aminotransferase, GGT = gamma-glutamine transpeptidase, TCH = total cholesterol, TG = triglyceride, LDL-C = low-density lipoprotein cholesterol, HDL-C = high-density lipoprotein cholesterol, FPG = fasting plasma glucose.

**Table 3:** Comparison of parameters between test group and control group after the 2-year lifestyle intervention.

Parameter	Control group	Test group	*P* value
	(n = 130)	(n = 130)	
Weight (kg)	75.97 ± 11.01	75.34 ± 9.94	0.627
SBP (mmHg)	120.38 ± 12.84	120.40 ± 13.70	0.993
DBP (mmHg)	78.99 ± 9.41	78.55 ± 11.70	0.735
FLI	52.06 ± 24.38	44.72 ± 23.08	0.013
NAFLD Fibrosis Score	−28.85 ± 2.12	−28.87 ± 2.27	0.939
**Liver Dysfunction**			
ALT (U/L)	28.78 ± 20.88	23.95 ± 12.50	0.025
AST (U/L)	23.56 ± 13.18	21.12 ± 6.90	0.063
GGT (mmol/L)	40.56 ± 32.88	33.96 ± 30.39	0.094
**Lipid profile**			
TCH (mmol/L)	4.86 ± 0.88	4.84 ± 0.82	0.878
LDL-C (mmol/L)	2.85 ± 0.67	2.92 ± 0.68	0.450
HDL-C (mmol/L)	1.23 ± 0.23	1.27 ± 0.26	0.193
TG (mmol/L)	2.07 ± 1.48	1.71 ± 0.93	0.022
**Insulin resistance**			
FPG (mmol/L)	5.03 ± 1.29	5.02 ± 1.08	0.988
**Liver Steatosis grade**			
Grade 0 n (%)	9 (6.9%)	41 (31.5%)	**<0.001**
Grade 1 n (%)	59 (45.4%)	69 (53.1%)	
Grade 2 n (%)	9 (6.9%)	12 (9.2%)	
Grade 3 n (%)	53 (40.8%)	8 (6.2%)	

FLI = fatty liver index, NAFLD-FS = NAFLD-fibrosis score, BMI = body mass index, SBP = systolic blood pressure, DBP = diastolic blood pressure, ALT = alanine aminotransferase, AST = aspartate aminotransferase, GGT = gamma-glutamine transpeptidase, TCH = total cholesterol, TG = triglyceride, LDL-C = low-density lipoprotein cholesterol, HDL-C = high-density lipoprotein cholesterol, FPG = fasting plasma glucose.

## References

[b1] McCullough A. J. The clinical features, diagnosis and natural history of nonalcoholic fatty liver disease. Clinics in liver disease 8, 521–533, viii, doi: 10.1016/j.cld.2004.04.004 (2004). 15331061

[b2] Rinella M. E. Nonalcoholic fatty liver disease: a systematic review. Jama 313, 2263–2273, doi: 10.1001/jama.2015.5370 (2015). 26057287

[b3] Setiawan V. W. . Prevalence of chronic liver disease and cirrhosis by underlying cause in understudied ethnic groups: The Multiethnic Cohort. Hepatology, doi: 10.1002/hep.28677 (2016). PMC5115980 27301913

[b4] Rahmani S. . Treatment of Non-alcoholic Fatty Liver Disease with Curcumin: A Randomized Placebo-controlled Trial. Phytotherapy research: PTR, doi: 10.1002/ptr.5659 (2016). 27270872

[b5] Chalasani N. . The diagnosis and management of non-alcoholic fatty liver disease: practice guideline by the American Gastroenterological Association, American Association for the Study of Liver Diseases, and American College of Gastroenterology. Gastroenterology 142, 1592–1609, doi: 10.1053/j.gastro.2012.04.001 (2012). 22656328

[b6] Adams L. A. . The natural history of nonalcoholic fatty liver disease: a population-based cohort study. Gastroenterology 129, 113–121 (2005). 16012941 10.1053/j.gastro.2005.04.014

[b7] Wong V. W. . Community-based lifestyle modification programme for non-alcoholic fatty liver disease: a randomized controlled trial. Journal of hepatology 59, 536–542, doi: 10.1016/j.jhep.2013.04.013 (2013). 23623998

[b8] Harrison S. A., Fecht W., Brunt E. M. & Neuschwander-Tetri B. A. Orlistat for overweight subjects with nonalcoholic steatohepatitis: A randomized, prospective trial. Hepatology 49, 80–86, doi: 10.1002/hep.22575 (2009). 19053049

[b9] Targher G., Day C. P. & Bonora E. Risk of cardiovascular disease in patients with nonalcoholic fatty liver disease. The New England journal of medicine 363, 1341–1350, doi: 10.1056/NEJMra0912063 (2010). 20879883

[b10] Orci L. A. . Exercise-based Interventions for Nonalcoholic Fatty Liver Disease: A Meta-analysis and Meta-regression. Clinical gastroenterology and hepatology: the official clinical practice journal of the American Gastroenterological Association, doi: 10.1016/j.cgh.2016.04.036 (2016). 27155553

[b11] Nobili V. . Lifestyle intervention and antioxidant therapy in children with nonalcoholic fatty liver disease: a randomized, controlled trial. Hepatology 48, 119–128, doi: 10.1002/hep.22336 (2008). 18537181

[b12] Boule N. G., Haddad E., Kenny G. P., Wells G. A. & Sigal R. J. Effects of exercise on glycemic control and body mass in type 2 diabetes mellitus: a meta-analysis of controlled clinical trials. Jama 286, 1218–1227 (2001). 11559268 10.1001/jama.286.10.1218

[b13] Umpierre D. . Physical activity advice only or structured exercise training and association with HbA1c levels in type 2 diabetes: a systematic review and meta-analysis. Jama 305, 1790–1799, doi: 10.1001/jama.2011.576 (2011). 21540423

[b14] Hu X. . Prevalence and factors associated with nonalcoholic fatty liver disease in Shanghai work-units. BMC gastroenterology 12, 123, doi: 10.1186/1471-230X-12-123 (2012). 22978800 PMC3499402

[b15] Johnson N. A. & George J. Fitness versus fatness: moving beyond weight loss in nonalcoholic fatty liver disease. Hepatology 52, 370–381, doi: 10.1002/hep.23711 (2010). 20578153

[b16] Hickman I. J. . Modest weight loss and physical activity in overweight patients with chronic liver disease results in sustained improvements in alanine aminotransferase, fasting insulin, and quality of life. Gut 53, 413–419 (2004). 14960526 10.1136/gut.2003.027581 PMC1773957

[b17] Saadeh S. . The utility of radiological imaging in nonalcoholic fatty liver disease. Gastroenterology 123, 745–750 (2002). 12198701 10.1053/gast.2002.35354

[b18] Dasarathy S. . Validity of real time ultrasound in the diagnosis of hepatic steatosis: a prospective study. Journal of hepatology 51, 1061–1067, doi: 10.1016/j.jhep.2009.09.001 (2009). 19846234 PMC6136148

[b19] Lin S. C. . Noninvasive Diagnosis of Nonalcoholic Fatty Liver Disease and Quantification of Liver Fat Using a New Quantitative Ultrasound Technique. Clinical gastroenterology and hepatology: the official clinical practice journal of the American Gastroenterological Association 13, 1337–1345 e1336, doi: 10.1016/j.cgh.2014.11.027 (2015). PMC4454635 25478922

[b20] Singh S. . Fibrosis progression in nonalcoholic fatty liver vs nonalcoholic steatohepatitis: a systematic review and meta-analysis of paired-biopsy studies. Clinical gastroenterology and hepatology: the official clinical practice journal of the American Gastroenterological Association 13, 643–654 e641–649; quiz e639-640, doi: 10.1016/j.cgh.2014.04.014 (2015). 24768810 PMC4208976

[b21] Reeder S. B., Cruite I., Hamilton G. & Sirlin C. B. Quantitative Assessment of Liver Fat with Magnetic Resonance Imaging and Spectroscopy. Journal of magnetic resonance imaging: JMRI 34, spcone, doi: 10.1002/jmri.22775 (2011). PMC3177109 22025886

[b22] Oh S. . Moderate to vigorous physical activity volume is an important factor for managing nonalcoholic fatty liver disease: a retrospective study. Hepatology 61, 1205–1215, doi: 10.1002/hep.27544 (2015). 25271091

[b23] Chalasani N. . The diagnosis and management of non-alcoholic fatty liver disease: practice Guideline by the American Association for the Study of Liver Diseases, American College of Gastroenterology, and the American Gastroenterological Association. Hepatology 55, 2005–2023, doi: 10.1002/hep.25762 (2012). 22488764

[b24] Koot B. G. . Intensive lifestyle treatment for non-alcoholic fatty liver disease in children with severe obesity: inpatient versus ambulatory treatment. International journal of obesity 40, 51–57, doi: 10.1038/ijo.2015.175 (2016). 26315844

[b25] Vilar-Gomez E. . Weight Loss Through Lifestyle Modification Significantly Reduces Features of Nonalcoholic Steatohepatitis. Gastroenterology 149, 367–378 e365; quiz e314-365, doi: 10.1053/j.gastro.2015.04.005 (2015). 25865049

[b26] Ryu S. . Relationship of sitting time and physical activity with non-alcoholic fatty liver disease. Journal of hepatology 63, 1229–1237, doi: 10.1016/j.jhep.2015.07.010 (2015). 26385766

[b27] Salomone F., Godos J. & Zelber-Sagi S. Natural antioxidants for non-alcoholic fatty liver disease: molecular targets and clinical perspectives. Liver international: official journal of the International Association for the Study of the Liver 36, 5–20, doi: 10.1111/liv.12975 (2016). 26436447

[b28] Marventano S. . Coffee and tea consumption in relation with non-alcoholic fatty liver and metabolic syndrome: A systematic review and meta-analysis of observational studies. Clinical nutrition, doi: 10.1016/j.clnu.2016.03.012 (2016). 27060021

[b29] Zelber-Sagi S. . Coffee consumption and nonalcoholic fatty liver onset: a prospective study in the general population. Translational research: the journal of laboratory and clinical medicine 165, 428–436, doi: 10.1016/j.trsl.2014.10.008 (2015). 25468486

[b30] Zelber-Sagi S., Godos J. & Salomone F. Lifestyle changes for the treatment of nonalcoholic fatty liver disease: a review of observational studies and intervention trials. Therapeutic advances in gastroenterology 9, 392–407, doi: 10.1177/1756283X16638830 (2016). 27134667 PMC4830109

[b31] Doycheva I. & Loomba R. Effect of metformin on ballooning degeneration in nonalcoholic steatohepatitis (NASH): when to use metformin in nonalcoholic fatty liver disease (NAFLD). Advances in therapy 31, 30–43, doi: 10.1007/s12325-013-0084-6 (2014). 24385405

[b32] Eslamparast T., Eghtesad S., Poustchi H. & Hekmatdoost A. Recent advances in dietary supplementation, in treating non-alcoholic fatty liver disease. World journal of hepatology 7, 204–212, doi: 10.4254/wjh.v7.i2.204 (2015). 25729475 PMC4342602

[b33] Eslamparast T., Eghtesad S., Hekmatdoost A. & Poustchi H. Probiotics and Nonalcoholic Fatty liver Disease. Middle East journal of digestive diseases 5, 129–136 (2013). 24829682 PMC3990183

[b34] Di Minno M. N. . Omega-3 fatty acids for the treatment of non-alcoholic fatty liver disease. World journal of gastroenterology 18, 5839–5847, doi: 10.3748/wjg.v18.i41.5839 (2012). 23139599 PMC3491590

